# Prevalence of Hypothyroidism Among Female Patients With Stress Urinary Incontinence Attending a Tertiary Hospital in Al-Baha, Saudi Arabia: A Retrospective Cross-Sectional Study

**DOI:** 10.7759/cureus.110350

**Published:** 2026-06-06

**Authors:** Ahmed Elaimeri, Amr Alemairy, Abeer Alemairy

**Affiliations:** 1 Department of Urology, King Fahad Hospital, Al-Baha, SAU; 2 Department of Internal Medicine, Community Medical Center, Toms River, USA; 3 Faculty of Medicine, University of Kassala, Kassala, SDN

**Keywords:** hypothyroidism, pelvic floor dysfunction, prevalence, saudi arabia, stress urinary incontinence, women's health

## Abstract

Background

Stress urinary incontinence (SUI) is a common pelvic floor disorder with significant physical and psychosocial impacts. Thyroid dysfunction may contribute to SUI by affecting skeletal muscle contractility, neuromuscular transmission, and connective tissue integrity. Limited research exists on this association among Saudi women. This study assessed the prevalence of hypothyroidism and its co-occurrence with clinical and demographic factors in women with clinically diagnosed SUI.

Methods

This retrospective cross-sectional study reviewed medical records of 288 women aged 20-50 years diagnosed with SUI at King Fahad Hospital, Al-Baha, Saudi Arabia (January 2020 to December 2025). Hypothyroidism was categorized as clinical (thyroid-stimulating hormone (TSH) >4.5 mIU/L with low free T4) or subclinical (TSH >4.5 mIU/L with normal free T4). Chi-square tests assessed associations with BMI, parity, diabetes, and hypertension. Age association was evaluated across three groups (20-29, 30-39, and 40-50 years) and by binary logistic regression. ORs with 95% CIs were calculated; p ≤ 0.05 was considered significant.

Results

The median age was 41 years (IQR: 12; range: 20-50 years); 54.2% of patients were aged 40-50 years. Hypothyroidism was identified in 136 patients (47.2%): 113 (39.2%) with clinical hypothyroidism and 23 (8.0%) with subclinical hypothyroidism. Significant associations were found with BMI (p = 0.003), parity (p = 0.005), and hypertension (p = 0.020); diabetes showed no significant association (p = 0.774). Hypothyroidism prevalence increased progressively with age: 33.3% (20-29 years), 43.8% (30-39 years), and 51.9% (40-50 years). Logistic regression confirmed a significant positive association between age and hypothyroidism (OR = 1.039 per year, 95% CI: 1.007-1.074, p = 0.019).

Conclusions

Hypothyroidism was highly prevalent among women with clinically diagnosed SUI and was frequently observed alongside higher BMI, multiparity, hypertension, and advancing age. These findings are hypothesis-generating; prospective interventional studies are needed before formal screening recommendations can be established.

## Introduction

Stress urinary incontinence (SUI) is one of the most prevalent pelvic floor disorders in women worldwide, defined by involuntary urine leakage during activities that increase intra-abdominal pressure, such as coughing, sneezing, or exercise. The condition significantly impairs physical function, emotional well-being, and quality of life. Epidemiological studies estimate that 25-45% of adult women globally experience urinary incontinence, with SUI representing the most common subtype, particularly among women of reproductive and middle age [1-6]. The pathophysiology of SUI involves dysfunction of pelvic floor support structures, urethral sphincter weakness, and neuromuscular injury, compounded by risk factors including multiparity, obesity, aging, and chronic disease [7-9].

Thyroid hormones regulate skeletal muscle metabolism, neuromuscular transmission, and connective tissue homeostasis. Hypothyroidism has been associated with reduced muscle contractility, impaired neuromuscular reflexes, glycosaminoglycan deposition in connective tissues, and collagen remodeling [10]. These alterations may compromise the structural and functional integrity of pelvic floor muscles and urethral support, potentially contributing to urinary continence disorders. Several studies have examined the relationship between thyroid dysfunction and lower urinary tract symptoms, including urinary incontinence, with mixed findings attributable to differences in study design, population characteristics, and diagnostic criteria [11-15].

Thyroid disorders are prevalent in Saudi Arabia, where hypothyroidism affects an estimated 10-30% of adult women [16-20]. Understanding this association may identify modifiable metabolic contributors to pelvic floor disorders and inform broader clinical evaluation strategies.

This study, therefore, aimed to determine the prevalence of hypothyroidism among women with clinically diagnosed SUI at King Fahad Hospital, Al-Baha, Saudi Arabia, and to assess associations between thyroid status and BMI, parity, diabetes mellitus, and hypertension within this population.

## Materials and methods

Study design and setting

This retrospective cross-sectional study was conducted at King Fahad Hospital, a tertiary referral institution serving the Al-Baha region of Saudi Arabia. Medical records from January 2020 to December 2025 were reviewed. The extended study period was selected to ensure an adequate sample size given the expected annual SUI case volume at this institution; no major changes in SUI diagnostic criteria or thyroid testing protocols were introduced during this period.

Study population

The study population comprised female patients aged 21-50 years with a clinically diagnosed SUI documented by a urology specialist during the study period. This age range focuses on women in the reproductive and early middle-age stages, where SUI is frequently encountered. Patient records were identified through the hospital medical record system. The study employed a total coverage sampling approach, including all eligible records within the defined period. Thyroid function test (TFT) results were available for all 288 women meeting the inclusion criteria during the study period. No eligible patients were excluded because of missing TFT data. The 288 patients, therefore, represent the complete eligible population identified within the defined period, not a selected subset.

Inclusion criteria

Female patients with a physician-documented clinical diagnosis of SUI and available TFT results in their medical records were eligible for inclusion.

Exclusion criteria

Patients with neurogenic bladder disorders, mixed urinary incontinence without a dominant stress component, or a history of pelvic floor or anti-incontinence surgery were excluded. Pregnant and postpartum women (within six months after delivery) were also excluded.

Data collection

Data were obtained through retrospective review of hospital medical records. Variables extracted included age, BMI, parity, duration of urinary symptoms, TFT results, and comorbid conditions. All data were anonymized. Available medical records did not include information on prior thyroid diagnoses or thyroid hormone replacement therapy. Thyroid status was classified solely based on the most recently documented thyroid-stimulating hormone (TSH) and free T4 values in the medical record at the time of SUI evaluation. Numerical TSH values were not consistently recorded in an extractable format across all records; thyroid status was therefore derived from physician-documented categorical diagnoses and binary free T4 classification.

Definition of thyroid status

Clinical hypothyroidism was defined as elevated TSH (> 4.5 mIU/L) with low free T4. Subclinical hypothyroidism was defined as elevated TSH (> 4.5 mIU/L) with normal free T4. Patients with normal TSH and free T4 levels were classified as euthyroid [21]. The TSH threshold of 4.5 mIU/L reflects the conventional 97.5th percentile reference range. It is acknowledged that TSH may be physiologically elevated in individuals with obesity independent of true thyroid dysfunction; given the high proportion of overweight and obese patients in this cohort (96.9%), some cases classified as subclinical hypothyroidism may represent BMI-related TSH elevation rather than intrinsic thyroid pathology. Furthermore, thyroid status was classified based on a single documented measurement; subclinical hypothyroidism ideally requires confirmation on repeat testing, as TSH may normalize spontaneously.

Statistical analysis

Categorical variables were summarized using frequencies and percentages. Age was non-normally distributed and is presented as median and IQR. The chi-square test was applied to evaluate associations between thyroid status and clinical variables. The association between age as a continuous variable and hypothyroidism was additionally evaluated using binary logistic regression, with results expressed as ORs with 95% CIs. A p-value of less than 0.05 was considered statistically significant. ORs with 95% CIs were calculated for binary variables. All analyses were performed using R version 4.5.2 (R Foundation for Statistical Computing, Vienna, Austria).

## Results

A total of 288 women were included, representing the complete eligible population identified at King Fahad Hospital during the defined study period (January 2020 to December 2025). The median age was 41 years (IQR: 12 years; range: 21-50 years). The majority of patients (54.2%, n = 156) were aged 40-50 years, followed by the 30-39-year group (36.5%, n = 105) and the 20-29-year group (9.4%, n = 27). Baseline characteristics are summarized in Table 1.

**Table 1:** Baseline characteristics of study participants (n = 288) Data are presented as frequency (n) and percentage (%). Age is presented as median (IQR); age groups were created using 10-year intervals. SUI, stress urinary incontinence

Variable	Category	n	%
Age (years)	Median 41 (IQR: 12; range: 21-50)	-	-
	20-29	27	9.4%
	30-39	105	36.5%
	40-50	156	54.2%
Thyroid status	Clinical hypothyroidism	113	39.2%
	Subclinical hypothyroidism	23	8.0%
	Total hypothyroid	136	47.2%
	Euthyroid	152	52.8%
BMI (kg/m²)	<25 (normal)	9	3.1%
	25-30 (overweight)	233	80.9%
	>30 (obese)	46	16.0%
Parity	0-2	36	12.5%
	3-5	197	68.4%
	>5	55	19.1%
Comorbidities	Diabetes mellitus	86	29.9%
	Hypertension	41	14.2%
Duration of SUI symptoms	<1 year	3	1.0%
	1-5 years	237	82.3%
	≥5 years	48	16.7%

**Figure 1:**
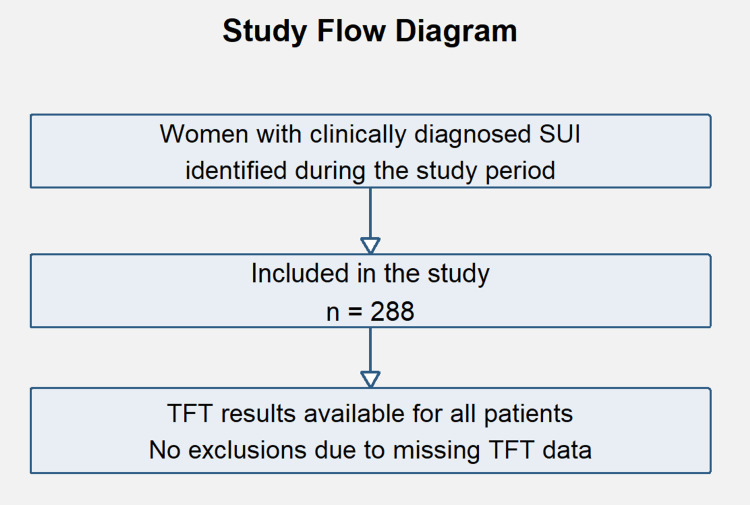
Study flow diagram of women with clinically diagnosed SUI included in the study SUI, stress urinary incontinence; TFT, thyroid function test

Figure 1 shows the study flow diagram of women with clinically diagnosed SUI included in the study. 

Hypothyroidism was identified in 136 patients (47.2%), including clinical hypothyroidism (n = 113; 39.2%), subclinical hypothyroidism (n = 23; 8.0%), and euthyroid status (n = 152; 52.8%).

No significant association was found with diabetes (χ² = 0.08, df = 1, p = 0.774; OR = 0.90, 95% CI: 0.54-1.49). A statistically significant inverse association was identified with hypertension (χ² = 5.37, df = 1, p = 0.020; OR = 0.41, 95% CI: 0.20-0.84), indicating that hypertension was less frequent among hypothyroid than euthyroid patients. Significant associations were also observed with BMI (χ² = 11.38, df = 2, p = 0.003) and parity (χ² = 10.76, df = 2, p = 0.005).

Regarding age, a stepwise increase in hypothyroidism prevalence was observed across age groups (20-29 years: 33.3%; 30-39 years: 43.8%; 40-50 years: 51.9%; χ² = 3.96, df = 2, p = 0.138). Although the chi-square analysis did not reach statistical significance, logistic regression confirmed a statistically significant positive association between continuous age and hypothyroidism (OR = 1.039 per year of age, 95% CI: 1.007-1.074, p = 0.019), indicating a 3.9% increase in the odds of hypothyroidism for each additional year of age.

The associations between hypothyroidism and clinical variables are summarized in Table 2.

**Table 2:** Association between hypothyroidism and clinical variables (n = 288) ^*^ Statistically significant at p < 0.05 Age association was tested by chi-square analysis across three age groups and confirmed by logistic regression (OR = 1.039 per year of age, 95% CI: 1.007-1.074, p = 0.019).

Variable	Category	Hypothyroid, n (%)	Euthyroid, n (%)	χ² (df)	p-value	OR (95% CI)
Age (years)	20-29	9 (33.3%)	18 (66.7%)	-	-	-
	30-39	46 (43.8%)	59 (56.2%)	-	-	-
	40-50	81 (51.9%)	75 (48.1%)	3.96 (2)	0.1378	-
	Overall (logistic OR = 1.039, 95% CI: 1.007-1.074, p = 0.019)	136 (47.2%)	152 (52.8%)	-	OR = 1.039; p = 0.019^*^	-
Diabetes mellitus	Yes	39 (28.7%)	47 (30.9%)	-	-	0.96 (0.58-1.59)
	No	97 (71.3%)	105 (69.1%)	0.08 (1)	0.774	Ref
Hypertension	Yes	12 (8.8%)	29 (19.1%)	-	-	0.41 (0.2-0.84)
	No	124 (91.2%)	123 (80.9%)	5.37 (1)	0.020^*^	Ref
BMI (kg/m²)	<25	0 (0.0%)	9 (5.9%)	-	-	-
	25-30	119 (87.5%)	114 (75.0%)	-	-	-
	>30	17 (12.5%)	29 (19.1%)	11.38 (2)	0.003^*^	-
Parity	0-2	9 (6.6%)	27 (17.8%)	-	-	-
	3-5	94 (69.1%)	103 (67.8%)	-	-	-
	>5	33 (24.3%)	22 (14.5%)	10.76 (2)	0.005^*^	-

A comparison of hypothyroidism prevalence across international studies is presented in Table 3.

**Table 3:** International comparison of hypothyroidism prevalence in women with urinary symptoms ^*^ For Can and Can (2025) [23], 65 women were included: 21 in the hypothyroid group, 16 in the subclinical hypothyroid group, and 28 controls. A statistically significant difference in the prevalence of SUI was observed only between the hypothyroid group and the control group, while there was no significant difference in SUI severity among the groups. Therefore, this study is best treated as a prospective controlled study of SUI rather than a prevalence study. SUI, stress urinary incontinence

Study	Country	n	Hypothyroidism prevalence (%)
Zargham et al. (2022) [2]	Iran	210	35.6
Løwenstein et al. (2021) [22]	Denmark	7,699	7.9
Can and Can (2025) [23]	Turkey	65	Not reported^*^
Present study	Saudi Arabia	288	47.2

**Figure 2:**
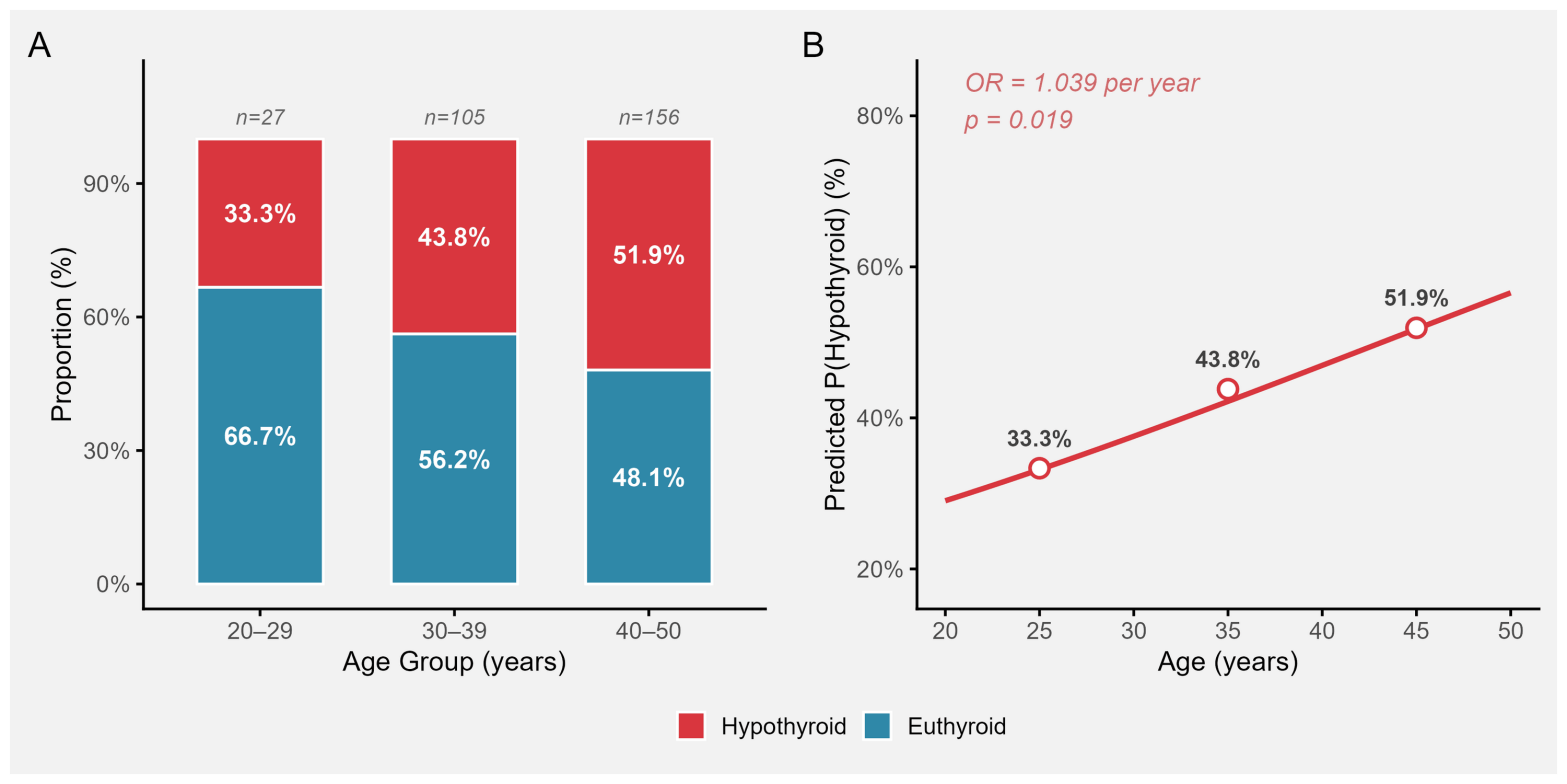
Association between age and hypothyroidism among women with SUI (n = 288) (A) Proportion of hypothyroid and euthyroid patients within each age group. (B) Logistic regression probability curve with observed group proportions overlaid. SUI, stress urinary incontinence

Figure 2 illustrates the association between age and hypothyroidism in this cohort using two complementary approaches. Panel A demonstrates a clear stepwise increase in hypothyroidism prevalence across the three age groups: 33.3% in women aged 20-29 years (n = 27), 43.8% in those aged 30-39 years (n = 105), and 51.9% in the 40-50-year group (n = 156). Panel B confirms this trend statistically through logistic regression, which revealed that each additional year of age was associated with a 3.9% increase in the odds of hypothyroidism (OR = 1.039, 95% CI: 1.007-1.074, p = 0.019). This age-related gradient is consistent with the known epidemiology of thyroid dysfunction in perimenopausal women.

**Figure 3:**
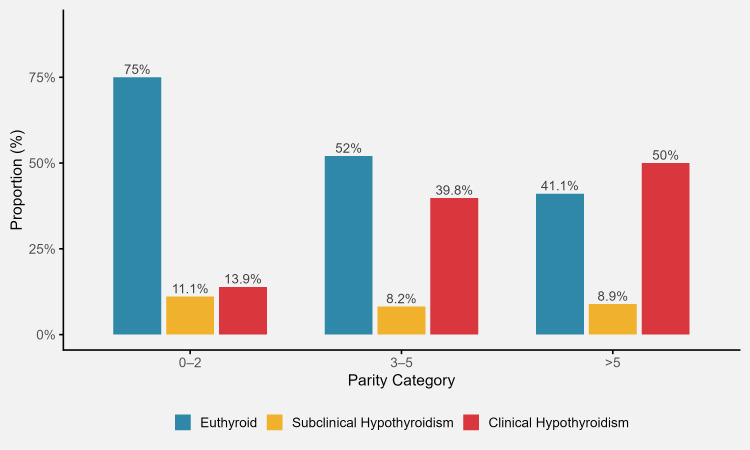
Distribution of thyroid status by parity category among women with SUI (n = 288) Values represent proportions within each parity group. Chi-square test: χ² = 10.76, df = 2, p = 0.005 SUI, stress urinary incontinence

Figure 3 displays the distribution of thyroid status stratified by parity category. Among women with low parity (0-2 deliveries), 75% were euthyroid, with clinical hypothyroidism accounting for only 13.9% of cases. This pattern reversed markedly with increasing parity: among women with more than five deliveries, clinical hypothyroidism represented 50% of cases, while the euthyroid proportion declined to 41.1%. The subclinical hypothyroidism proportion remained consistently low across all parity groups (8.2-11.1%), suggesting that the parity effect is primarily driven by clinical rather than subclinical disease.

**Figure 4:**
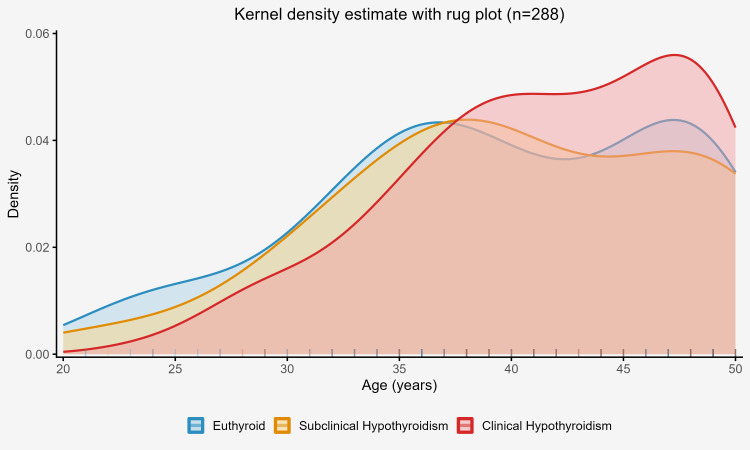
Kernel density estimates of age distribution stratified by thyroid status among women with SUI (n = 288) The rug plot at the base indicates individual patient ages. SUI, stress urinary incontinence

The continuous age distribution across thyroid status groups is further illustrated in Figure 4. The clinical hypothyroidism group exhibited a distinctly right-shifted density curve with a pronounced peak between ages 45 and 50 years, contrasting with the euthyroid group, which showed a broader, more symmetric distribution peaking around age 43 years. The subclinical hypothyroidism group displayed an intermediate pattern. The rug plot confirms the sparse representation of younger women (20-29 years) across all three groups, consistent with the lower overall prevalence observed in the 20-29-year age band. Together, Figure 2 and Figure 4 provide convergent visual evidence that age is a meaningful correlate of hypothyroidism severity in this cohort.

## Discussion

This study found a hypothyroidism prevalence of 47.2% among women with clinically diagnosed SUI attending a single tertiary center. As the study lacks a non-SUI comparator group, this figure describes the within-cohort prevalence only and cannot be interpreted as evidence that hypothyroidism is more common in women with SUI than in the general population. Nonetheless, this prevalence substantially exceeds the 5-15% range reported in general female population surveys. Saudi epidemiological data report 10-30% hypothyroidism among adult women [16-21,24,25]. International studies reported 18-31% prevalence in women with urinary symptoms [2,22,23]. Thyroid hormones play a central role in skeletal muscle metabolism and neuromuscular transmission [10-15]. Hypothyroidism is associated with reduced muscle contractility and myopathic changes [26]. Parity demonstrated a significant association, consistent with cumulative pelvic floor trauma from repeated vaginal deliveries [7-9,26].

The positive association between advancing age and hypothyroidism (OR = 1.039 per year, 95% CI: 1.007-1.074, p = 0.019) is consistent with established evidence that thyroid hormone deficiency induces progressive metabolic impairment and declining skeletal muscle contractility that accumulates with age [24,25]. In this cohort, 54.2% of patients were aged 40-50 years, the group with the highest hypothyroidism prevalence (51.9%), likely reflecting the convergence of thyroid autoimmunity and declining ovarian estrogen in perimenopausal women. The chi-square test across age groups did not reach significance (p = 0.138), attributable to limited power in the youngest group (n = 27); logistic regression using continuous age provided greater sensitivity and confirmed the association. Future prospective studies with age-stratified recruitment are needed to better characterize this relationship.

Although hypertension demonstrated a statistically significant association with hypothyroidism (p = 0.020), the observed OR was less than 1 (OR = 0.41), indicating that hypertension was less frequent among hypothyroid patients. Physiologically, hypothyroidism is associated with diastolic hypertension through increased peripheral vascular resistance; therefore, higher rates would be expected [27]. Several explanations are plausible: antihypertensive medication use may have masked true differences; differential healthcare utilization patterns may result in underdocumentation of hypertension; and sampling variation in a single-center cohort may explain the finding. Data on antihypertensive medication use were not available in the medical records. The association should be interpreted with caution and verified in larger prospective studies.

Diabetes mellitus showed no significant association (p = 0.774). Residual confounders, including menopausal status, delivery mode, smoking, and thyroid treatment history, could not be evaluated because of the retrospective design. Limitations include the single-center setting and the unavailability of key clinical variables. It is acknowledged that in other clinical settings, TFTs may be preferentially ordered for women with multiple comorbidities or thyroid-related symptoms; such selective testing would be expected to overestimate true prevalence. At this hospital, TFTs were available for all 288 eligible patients, mitigating this specific concern. Future prospective multicenter studies with standardized thyroid testing for all SUI patients are needed.

Thyroid medication history was not available in the medical records, and the inclusion of treated patients may therefore have led to overestimation of prevalence. The study also lacked a non-SUI comparator group, precluding comparison with women without SUI. Finally, subclinical hypothyroidism was defined on the basis of a single TSH measurement, which may normalize on repeat testing.

## Conclusions

This retrospective cross-sectional study found that hypothyroidism was prevalent in 47.2% of women with clinically diagnosed SUI attending a tertiary hospital in Al-Baha, Saudi Arabia. Within this cohort, hypothyroidism was frequently observed alongside higher BMI, multiparity, and advancing age. An inverse association with hypertension was identified but should be interpreted with caution given the absence of thyroid medication history data. As this study lacks a comparator group and is retrospective in design, causal inference and population-level generalization cannot be established. These findings are hypothesis-generating and support future prospective multicenter studies with a non-SUI control arm, standardized thyroid testing, and documentation of thyroid treatment history to confirm the clinical relevance of these observations.

## References

[1] Tahra A, Bayrak Ö, Dmochowski R (2022). The epidemiology and population-based studies of women with lower urinary tract symptoms: a systematic review. Turk J Urol.

[2] Zargham M, Hajian MR, Alizadeh F, Eslami MJ, Khalili Boroujeni N, Gholipour F (2022). Hypothyroidism is prevalent among adult women with chronic lower urinary tract symptoms. Low Urin Tract Symptoms.

[3] Nygaard I, Barber MD, Burgio KL (2008). Prevalence of symptomatic pelvic floor disorders in US women. JAMA.

[4] Peinado-Molina RA, Hernández-Martínez A, Martínez-Vázquez S, Rodríguez-Almagro J, Martínez-Galiano JM (2023). Pelvic floor dysfunction: prevalence and associated factors. BMC Public Health.

[5] ShahAli S, Bø K, Hejazi A, Hashemi H, Kharaji G (2025). Effect of pelvic floor muscle training on pelvic floor muscle morphometry in subjects with pelvic organ prolapse: a systematic review and meta-analysis. BMC Womens Health.

[6] Subak LL, Richter HE, Hunskaar S (2009). Obesity and urinary incontinence: epidemiology and clinical research update. J Urol.

[7] Bø K (2003). Pelvic floor muscle strength and response to pelvic floor muscle training for stress urinary incontinence. Neurourol Urodyn.

[8] Moris L, Heesakkers J, Nitti V (2025). Prevalence, diagnosis, and management of stress urinary incontinence in women: a collaborative review. Eur Urol.

[9] Zhang RQ, Xia MC, Cui F, Chen JW, Bian XD, Xie HJ, Shuang WB (2021). Epidemiological survey of adult female stress urinary incontinence. BMC Womens Health.

[10] Gabrielson AT, Sartor RA, Hellstrom WJ (2019). The impact of thyroid disease on sexual dysfunction in men and women. Sex Med Rev.

[11] Razvi S, Jabbar A, Pingitore A (2018). Thyroid hormones and cardiovascular function and diseases. J Am Coll Cardiol.

[12] Burzynski B, Jurys T, Knapik M, Burzynski K, Rzymski P, Rajwa P, Bryniarski P (2023). Abdominal complex muscle in women with stress urinary incontinence - prospective case-control study. Arch Med Sci.

[13] Rostami P, Mostafaei H, Salehi-Pourmehr H, Hajebrahimi S (2025). An updated systematic review and meta-analysis on the prevalence of female urinary incontinence in developing countries—a collaborative report by the International Continence Society (Developing World Committee) and the Iranian Research Center for Evidence-Based Medicine. Neurourol Urodyn.

[14] Vahdatpour B, Zargham M, Chatraei M, Bahrami F, Alizadeh F (2015). Potential risk factors associated with stress urinary incontinence among Iranian women. Adv Biomed Res.

[15] Yang X, Wang X, Gao Z (2022). The anatomical pathogenesis of stress urinary incontinence in women. Medicina (Kaunas).

[16] Alqahtiani NM, Alramadhan ZT, Kurdi AN (2020). Hypothyroidism in Saudi Arabia; prevalence, risk factors, and its relation with diabetes mellitus. Arch Pharm Pract.

[17] Al Shahrani AS, El-Metwally A, Al-Surimi K (2016). The epidemiology of thyroid diseases in the Arab world: a systematic review. J Public Health Epidemiol.

[18] Aldossari K, Al-Ghamdi S, Al-Zahrani J, Al Jammah A, Alanazi B, Al-Briek A, Alanazi M (2019). Association between subclinical hypothyroidism and metabolic disorders: a retrospective chart review study in an emerging university hospital. J Clin Lab Anal.

[19] Elmisbah HO, Laham RY, Almisbah HO (2024). Prevalence of thyroid disorders among the diabetic population in Arar, Saudi Arabia. Saudi Med J.

[20] Saeed MI, Hassan AA, Butt ME (2018). Pattern of thyroid lesions in western region of Saudi Arabia: a retrospective analysis and literature review. J Clin Med Res.

[21] Baretella O, Blum MR, Abolhassani N (2025). Associations between subclinical thyroid dysfunction and cardiovascular risk factors according to age and sex. J Clin Endocrinol Metab.

[22] Løwenstein E, Jepsen R, Andersen LL (2021). Hypothyroidism and urinary incontinence: prevalence and association in a Danish, female sample from the Lolland-Falster Health study. Eur J Obstet Gynecol Reprod Biol.

[23] Can B, Can O (2025). The relationship between hypothyroidism and stress urinary incontinence: a prospective controlled study. Med Bull Haseki.

[24] Teixeira PF, Dos Santos PB, Pazos-Moura CC (2020). The role of thyroid hormone in metabolism and metabolic syndrome. Ther Adv Endocrinol Metab.

[25] Duntas LH, Brenta G (2012). The effect of thyroid disorders on lipid levels and metabolism. Med Clin North Am.

[26] Hunskaar S, Arnold EP, Burgio K, Diokno AC, Herzog AR, Mallett VT (2000). Epidemiology and natural history of urinary incontinence. Int Urogynecol J Pelvic Floor Dysfunct.

[27] Klein I, Danzi S (2007). Thyroid disease and the heart. Circulation.

